# A novel method for interrogating receiver operating characteristic curves for assessing prognostic tests

**DOI:** 10.1186/s41512-017-0017-y

**Published:** 2017-11-15

**Authors:** Grégoire Thomas, Louise C. Kenny, Philip N. Baker, Robin Tuytten

**Affiliations:** 1 SQU4RE, 8800 Roeselare, Belgium; 2 Department of Obstetrics and Gynaecology, University College Cork, Cork, Ireland; 3 College of Medicine, Biological Sciences and Psychology, University of Leicester, University Road, Leicester, LE1 7RH UK; 4 Metabolomic Diagnostics, Hoffmann Park, Little Island, Cork, Ireland; 5 Irish Centre for Fetal and Neonatal Translational Research (INFANT), 5th Floor, Cork University Maternity Hospital, Cork, Ireland

**Keywords:** Prognosis, Biomarkers, Multi-component tests, Prognostic performance

## Abstract

**Background:**

Disease prevalence is rarely explicitly considered in the early stages of the development of novel prognostic tests. Rather, researchers use the area under the receiver operating characteristic (AUROC) as the key metric to gauge and report predictive performance ability. Because this statistic does not account for disease prevalence, proposed tests may not appropriately address clinical requirements. This ultimately impedes the translation of prognostic tests into clinical practice.

**Methods:**

A method to express positive- and/or negative predictive value criteria (PPV, NPV) within the ROC space is presented. Equations are derived for so-called equi-PPV (and equi-NPV) lines. Herewith it is possible, for any given prevalence, to plot a series of sensitivity-specificity pairs which meet a specified PPV (or NPV) criterion onto the ROC space.

This concept is introduced by firstly reviewing the well-established “mechanics”, strengths and limitations of the ROC analysis in the context of developing prognostic models. Then, the use of PPV (and/or) NPV criteria to augment the ROC analysis is elaborated.

Additionally, an interactive web tool was also created to enable people to explore the dynamics of lines of equi-predictive value in function of prevalence. The web tool also allows to gauge what ROC curve shapes best meet specific positive and/or negative predictive value criteria (http://d4ta.link/ppvnpv/).

**Results:**

To illustrate the merits and implications of this concept, an example on the prediction of pre-eclampsia risk in low-risk nulliparous pregnancies is elaborated.

**Conclusions:**

In risk stratification, the clinical usefulness of a prognostic test can be expressed in positive- and negative predictive value criteria; the development of novel prognostic tests will be facilitated by the possibility to co-visualise such criteria together with ROC curves. To achieve clinically meaningful risk stratification, the development of separate tests to meet either a pre-specified positive value (rule-in) or a negative predictive value (rule-out) criteria should be considered: the characteristics of successful rule-in and rule-out tests may markedly differ.

**Electronic supplementary material:**

The online version of this article (10.1186/s41512-017-0017-y) contains supplementary material, which is available to authorized users.

## Background

With the increasing availability of high-throughput platforms and technologies capable of exploring the entire “omics” pipeline, contemporary biomarker discovery studies often yield extensive lists of putative biomarkers. Herewith the simultaneous development and/or evaluation of various prognostic test permutations becomes conceivable. By combining specific subsets of markers, as determined in a single “omics” analysis, different prognostic paradigms can potentially be explored and a variety of clinical perspectives can simultaneously be accommodated.

However, during our efforts to leverage this modulation potential of “omics” in the development of novel prognostic tests for pre-eclampsia [1, 2], we were confronted with a “missing link” when it came to defining prognostic test performance specifications. Where clinical practitioners will often gauge the merits of a test in terms of prevalence-dependent metrics like positive predictive value (PPV) or negative predictive value (NPV), test developers will usually use other statistics, such as the area under the receiver operating characteristic (AUROC, also referred to as the *c*-statistic or the AUC), which are considered prevalence independent, to do the same. Here, we present a method which seamlessly links these two views upon prognostic test performances: the ability to plot PPV or NPV criteria, which account for prevalence, in the receiver operating characteristic (ROC) space. To illustrate the merits and implications of this concept, we use the prediction of pre-eclampsia risk.

### AUROC: popular tool for evaluating prognostic tests

Statistics like sensitivity (*S*_*n*_), specificity (*S*_*p*_) and the AUROC remain widely employed in the development and assessment of prognostic tests, whereby “prognosis relates to the probability or risk of an individual developing a particular state of health (an outcome) over a specific time” [quoted from Moons et al. [3]]. This is especially true in biomarker discovery research and the early stages of translational research, where *S*_*n*_, *S*_*p*_ and the AUROC are commonly considered independent of the underlying prevalence of the condition under study. Albeit it is known that differences in patient spectrum lead to test performance variation across different population subgroups [4], the assumed independence of *S*_*n*_, *S*_*p*_ and the AUROC facilitates the use of cost-effective case-control studies to evaluate the merits of possible novel prognostic markers or tests [5].

The AUROC, essentially a measure of discrimination, corresponds to the probability that a classifier will correctly rank a randomly chosen person with the condition higher than a randomly chosen person without the condition [4]. The AUROC may not be optimal in assessing prognostic models or models that stratify individuals into risk categories [6]. In this setting, model calibration (a measure of how well predicted probabilities agree with actual observed risk) is also important for the accurate assessment of risk [7]. Furthermore, since the AUROC is not a function of the actual predicted probabilities but is based solely on ranks, its use for model selection could possibly eliminate useful risk factors from prediction scores [8]. Notwithstanding the fact that the above limitations of the AUROC in evaluating prognostic models are well established [8, 9], the AUROC remains widely used to report on prognostic model development efforts, and there is a continuing reliance on the AUROC to evaluate novel and emerging risk factors and biomarkers.

At the same time, the convenience of being largely independent of disease prevalence is also the key limitation of the use of the AUROC in prognostic test development. Clinical decisions and access to certain clinical care pathways are mostly governed by weighing the benefits versus the costs at the level of the intended-use population. For a so-called “rule-in” test, the benefit of the early detection of risk in those who will develop the disease (true positives) needs to be balanced against the cost of wrongly identifying individuals as being at high risk (false positives). Vice versa, for a “rule-out” test, the benefits of finding true negatives will be weighed against wrongly identifying false negatives as being at low risk. When a prognostic test is assessed in its clinically relevant context, metrics like positive and negative predictive values (PPV and NPV), which take the disease prevalence into account, are more appropriate [10].

## Methods

### Prognostic tests: AUROC, ROC curves and thresholds

**Fig. 1:**
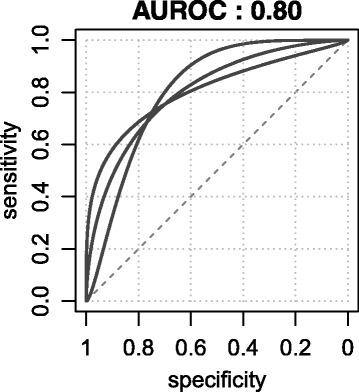
Three different receiving operating curves with the same AUROC

The ROC curve follows the calculation of sensitivity and specificity for all the test values obtained within a study; sensitivity is plotted against 1-specificity in a ROC curve (Fig. 1). Sensitivity (*S*_*n*_) is equal to the true positive rate and is expressed in function of true positives (TP) and false negatives (FN) as follows:

$$ {S}_n=\frac{\mathrm{TP}}{\mathrm{TP}+\mathrm{FN}}\kern2.5em (1) $$

Specificity (*S*_*p*_) is equal to the true negative rate and is classically expressed in function of true negatives (TN) and false positives (FP) as follows:

$$ {S}_p=\frac{\mathrm{TN}}{\mathrm{TN}+\mathrm{FP}}\kern2.5em (2) $$

The AUROC is considered a measure of the performance of a prognostic test, ranging from an area of 0.5 (non-discriminative test, the diagonal) up to 1 (a perfect test with perfect discrimination of future cases and controls). It is obvious from Fig. 1 that for a given AUROC, differently shaped ROC curves can be found, whereby each different shape corresponds to a prognostic test with different prediction characteristics.

**Fig. 2:**
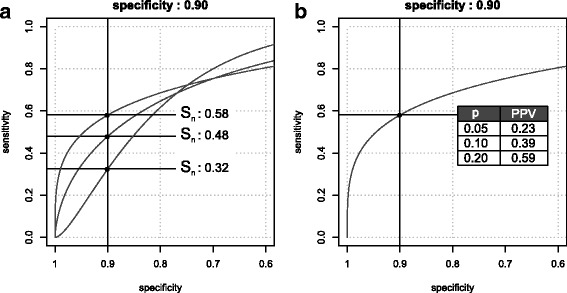
Sensitivity and PPV at a given specificity. **a** Sensitivity at a given specificity (*S*_*p*_ = 0.90) for three ROC curves with the same AUROC (full ROC curves shown in Fig. 1). **b** PPV as a function of disease prevalence (*p =* 0.05, 0.10, 0.20) for given specificity (*S*_*p*_ = 0.90) and sensitivity (*S*_*n*_ = 0.58)

When a dichotomous test is required, a threshold for the score is defined. For instance, to identify a population at risk, it is common to lock the false positive rate (FPR) allowed and then to observe where the ROC curve crosses the specificity criterion [11, 12]. In Fig. 2a, it is shown that for three differently shaped ROC curves, yet with the same AUROC, this criterion results in three different sensitivities.

As mentioned earlier, the statistics AUROC, *S*_*n*_ and *S*_*p*_ are considered prevalence-independent statistics [13], yet prevalence is important when assessing the clinical usefulness of a prognostic test [14]. In case of a low prevalence disease, high sensitivity and specificity can still be associated with (very) low PPVs.

Prognostic test performance assessments should therefore also consider metrics that take the prevalence of a disease such as PPV into account, i.e. the fraction of patients that will actually develop the condition (TP) within the group of all patients that have a positive test (TP + FP). Fig. 2b, illustrates how, for the same specificity threshold, prevalence modulates the PPV achieved. Applying Bayes’ theorem, PPV can be expressed in terms of *S*_*n*_, *S*_*p*_ and prevalence *p* [15]:

$$ \mathrm{PPV}=\frac{S_n\ p}{S_n\kern0.5em p+\left(1-{S}_p\right)\ \left(1-p\right)}\kern2.5em (3) $$

In a similar fashion, one can show a linear relationship between the multiplicative inverse of PPV, prevalence and positive likelihood ratio; Additional file 1: equations 3’ and 3”.

Therefore, and as shown in Fig. 2b, the PPV increases with prevalence for a fixed sensitivity and specificity (or fixed likelihood ratio).

Moreover, this illustrates that the utility of a prognostic test cannot be determined by merely estimating whether its sensitivity and/or specificity are higher than or equal to a predefined cut-off. Indeed, a lower specificity is permissible if sensitivity is higher.

**Fig. 3:**
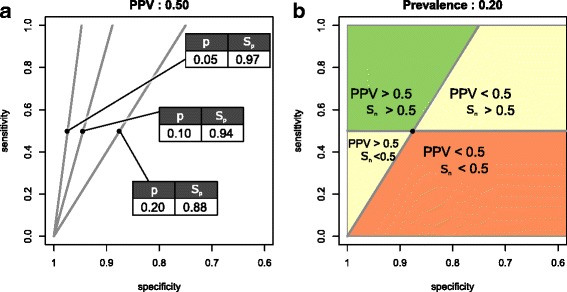
Illustrations of positive predictive value thresholds. **a** Impact of prevalence: equi-PPV lines for fixed positive predictive value (PPV = 0.50) and three different prevalence values; i.e. 0.05, 0.1 and 0.2. **b** Defining minimum predictive performance: division of the “ROC space” into four quadrants as defined by a PPV cut-off (PPV ≥ 0.50) and a sensitivity cut-off (*S*_*n*_ ≥ 0.50) corresponding minimum test requirements for a test to deliver clinically relevant prognostic performance (hypothetical). Only tests whose ROC curves cross both cut-off lines and have points in the upper-left quadrant (green) do outperform the minimum test requirements

Typically, a prognostic rule-in test should (1) identify a minimal proportion of the patients that will actually develop the disease and (2) ensure that this true positive group has a sufficiently large proportion of the patients testing positive. In other words, such prognostic tests must reach a minimal sensitivity and minimal PPV (Fig. 3b).

Likewise, a prognostic rule-out test should (1) identify a minimal proportion of patients that will certainly not develop the disease and (2) ensure that of the patients testing negative, sufficiently few will develop the disease (false negatives). Such test must therefore reach a minimal specificity and minimal negative predictive value (NPV); following Bayes’ theorem [14], NPV can be written as follows (Eq. (4)):

$$ \mathrm{NPV}=\frac{S_p\ \left(1-p\right)}{\left(1-{S}_n\right)\ p+{S}_p\ \left(1-p\right)}\kern2.5em (4) $$

As for PPV, a linear relationship between the multiplicative inverse of NPV, prevalence and negative likelihood ratio can be derived; Additional file 1: equations 4’and 4”.

### Equi-PPV and equi-NPV lines

When developing prognostic models for application in healthcare, preferentially the clinical context of the tests should be taken into account from the start. At the same time, the convenience of using cost-efficient case-control study designs and the well-established AUROCs to evaluate models in development is desirable. Presented with this conundrum, we established a means to visualise PPV (and NPV) criteria in the ROC space.

For a rule-in test, a clinically relevant minimal PPV and sensitivity are established. We can, for example, consider a hypothetical prognostic test which becomes clinically relevant when PPV ≥ 0.50 and sensitivity ≥ 0.50 (Fig. 3b). By rearranging the Eq. (3), it is possible to derive specificity (*S*_*p*_) in terms of sensitivity (*S*_*n*_) and PPV cut-off (PPV_c_):

$$ {S}_p=1-{S}_n\kern0.5em \frac{p}{\left(1-p\right)}\ \frac{\left(1-{\mathrm{PPV}}_{\mathrm{c}}\right)}{{\mathrm{PPV}}_{\mathrm{c}}}\kern2.5em (5) $$

whereby PPV_c_ is a fixed target value, and *S*_*n*_ is varied between 0 and 1. For a given prevalence, the specificity at which the PPV criterion is met can be calculated for each sensitivity (Fig. 3a). This series of sensitivities and specificities can be represented as a line onto a ROC plot: we call this line the *equi-PPV line* (Fig. 3). The equation for the equi-PPV line is:

$$ {S}_n=\left(1-{S}_p\right)\ \frac{\ \left(1-p\right)}{p}\kern0.5em \frac{{\mathrm{PPV}}_{\mathrm{c}}}{\left(1-{\mathrm{PPV}}_{\mathrm{c}}\right)}\kern2.75em (6) $$

Similarly, for a rule-out test an equi-NPV line can be derived and plotted on ROC plots (Eq. (7)):

$$ {S}_n=1-\kern0.5em {S}_p\kern0.5em \frac{\left(1-p\right)\ }{p}\kern0.5em \frac{\left(1-{\mathrm{NPV}}_{\mathrm{c}}\right)}{{\mathrm{NPV}}_{\mathrm{c}}}\kern2.75em (7) $$

where NPV_c_ is the NPV cut-off. This line corresponds the minimal NPV required to achieve clinical relevance.

As shown in Fig. 3, equi-PPV (equi-NPV) lines can be plotted in the ROC space. Combined with a sensitivity (specificity) target, they divide the ROC space into quadrants that correspond with the clinical relevance of a test. The predictive performance of a prognostic test can, therefore, be quickly estimated. If the ROC curve passes through the upper left quadrant, the test complies with the predetermined performance criteria.

### Software tool

To allow for the exploration of the relationship between the AUROC, sensitivity, specificity, prevalence and predictive values, a software tool was developed. Its dynamic interface permits the reader to gain an understanding in the dynamics of these relationship. The tool is available at the following address: http://d4ta.link/ppvnpv/. On this website, an R package is also made available so that the reader can perform PPV and NPV analyses on their own data.

## Results

### Developing a pre-eclampsia test for first-time pregnant women

We have a longstanding research interest in the prediction of pre-eclampsia risks in nulliparous women early in pregnancy using novel protein or metabolite biomarkers [1, 2]. First-time pregnant women have a risk of ~ 1/20 to develop pre-eclampsia [16], or a relative risk of approximately 2, compared to non-nulliparous [17].

In our continuous efforts to develop a clinically meaningful screening test, we recently proposed the following rationale [18]. The prenatal management of a multiparous woman with regards to pre-eclampsia is largely guided by her previous pregnancy history. Epidemiological studies have shown that previous pre-eclampsia is associated with an increased risk of recurrence. For a second pregnancy, recurrence risks of about 1 in 8.6 to 1 in 6.8 (or PPV of 0.116 to 0.147) are reported [19, 20], whereas a woman without prior pre-eclampsia will have a lower risk of 1 in 77 to 1 in 100 (or NPV of 0.987 to 0.99) [19, 20]. In line with this, if a woman has experienced pre-eclampsia in a previous pregnancy, she will be managed more vigilantly in most healthcare systems in high-resource settings, with more prenatal visits compared to a woman who did not develop pre-eclampsia in any earlier pregnancy.

Based on the above, we proposed that a pre-eclampsia risk stratification test for nulliparous should ideally mimic the pre-eclampsia risk information as available for a second-time pregnant woman. Therefore, the test should either stratify nulliparous women to a high-risk group with a post-test pre-eclampsia probability of at least 1 in 7.5 (equivalent to a PPV = 0.133; rule-in) or stratify them to a low-risk group with a post-test probability of at least 1 in 90 (equivalent to a NPV = 0.988; rule-out) and ideally both.

**Fig. 4:**
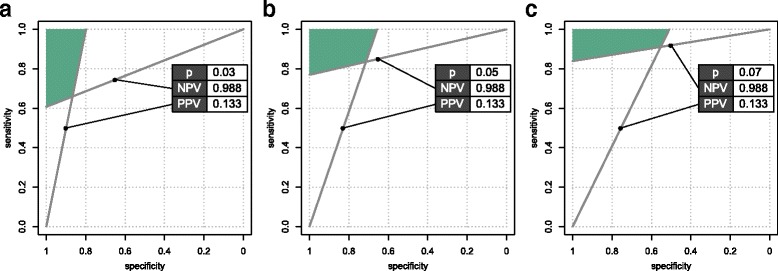
**a**–**c** Equi-PPV and equi-NPV lines corresponding the pre-eclampsia risk in multiparous pregnant women with previous pre-eclampsia (PPV) or without previous pre-eclampsia (NPV). These minimal prognostic performance thresholds are calculated for three different pre-eclampsia prevalence values, as reported for first-time pregnant women

In Fig. 4, we plotted both the proposed minimal PPV and NPV criteria on the ROC space to identify the quadrant in the ROC space which would comply with both these criteria simultaneously. To illustrate the impact of prevalence, the criteria for three published prevalence values were plotted: 0.05 [16], 0.03 [21], and 0.07 [22] (rounded for convenience).

The reader can appreciate that to achieve the success quadrant in each of the possible prevalence scenarios, a screening test with extraordinary *S*_*n*_ and *S*_*p*_ is required. The existence of such a test is unlikely; for instance, Royston et al. noted that the AUROC of prognostic models is typically between 0.6 and 0.85 [23]. Knowing that pre-eclampsia is a syndrome [24] that at time of risk prediction the future disease status remains to be determined by a stochastic process, the target population concerns healthy first-time pregnant women without any overt risk factors, and pre-eclampsia diagnoses cannot be made unequivocally [25], the failure to develop such a test should not be surprising. Yet the American College of Obstetricians and Gynecologists (ACOG) published recently that “useful prediction for pre-eclampsia would require a high likelihood ratio (greater than 10) for a positive test as well as a low likelihood for a negative result (less than 0.2)” [26]; one can calculate this would require for a prognostic test with a minimum *S*_*n*_ of 0.82, and associated *S*_*p*_ of 0.92, or AUROC ≥ 0.87.

Upon the realisation that a single prognostic test for pre-eclampsia in low-risk first-time pregnant women will not be able to meet the earlier proposed target PPV and NPV criteria, we investigated whether there are alternative ways to develop a clinically meaningful pre-eclampsia risk prediction tests for this intended patient population and how “omics” data could help achieve this.

**Fig. 5:**
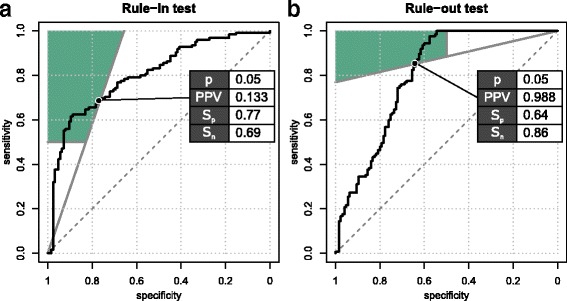
Multivariable modelling**.** Two possible prognostic test permutations for pre-eclampsia prediction in first-time pregnant women are shown (hypothetical data) (**a**). Rule-in test compliant with pre-set test specification: PPV ≥ 0.133, *S*_*n*_ ≥ 0.50, for a 5% disease prevalence. **b** Rule-out test compliant with pre-set test specification: NPV ≥ 0.988, *S*_*p*_ ≥ 0.50, for a 5% disease prevalence

We hypothesise that possibly more meaningful pre-eclampsia risk prediction can be achieved when the risk stratification question is resolved in its two constituting requirements: i.e., treat the rule-in and rule-out independently. Instead of a pursuing a single risk stratification test which meets both clinical PPV and NPV requisites, the development of separate rule-out and rule-in tests which complement each other and which can be deployed together, should be considered. To this end, minimal performance criteria for both tests must be established: for instance, for the rule-in test, it could be specified that at least 50% of all the cases need to be identified (*S*_*n*_ ≥ 0.50), similarly it could be specified that at least 50% of the non-cases need to be ruled out (*S*_*p*_ ≥ 0.50). The sections of the ROC space where these minimal performance criteria are met are highlighted in Fig. 5, based on the middle prevalence scenario (*p* = 0.05), please note the presented data are hypothetical.

## Discussion

The ability to plot PPV (and/or NPV) criteria in the ROC space provides the prognostic test developer with an informative tool as it allows for the explicit accounting for prevalence (pre-test probability) and the clinically desirable (or relevant) post-test probability. This is particularly relevant when developing and evaluating prognostic tests for diseases of low prevalence.

Prognostic tests often combine multiple variables to predict outcomes. The development of such multi-component tests involves the selection and optimization of a modelling technique and the selection of the relevant variables. In the test development phase, this process often focuses on maximising the AUROC only, irrespective of the underlying distribution of risk scores in cases and controls. In this phase, case control design is typically applied for practical and economic reasons; novel technologies (like “omics”) to discover or evaluate novel predictive markers are often cost and time intensive. Then, if a dichotomous test is pursued, a suitable cut-off needs to be selected: popular ways of selecting an optimal threshold include finding the point on the curve closest to the coordinate (*x* = 0, *y* = 1), and calculation of the Youden index [14, 27]. These methods give equal weight to sensitivity and specificity but do not consider disease prevalence. Consequently, when developing novel prognostic tests, all too often little thought is given to which predictive performance criteria are relevant to a specific clinical demand: ultimately, a test result should assist a clinician, to make an actionable decision. By solely relying on metrics such as the AUROC, sensitivity or specificity one risks selecting sub-optimal variables and models and ultimately proposing clinically meaningless tests.

In risk stratification, the aim is often to identify a population at increased risk (rule-in), or at decreased risk (rule-out), and to change care regimen accordingly. In this context, PPV and NPV are important determinants of the predictive performance of prognostic tests. Explicit consideration of minimal PPV and NPV criteria in test development bears the potential to deliver prognostic models which are more fit-for-purpose. For instance, in the pre-eclampsia example, the quoted pre-eclampsia prevalence values all related to low-risk nulliparous women (same care setting), yet the various populations exhibited different a priori risks. As illustrated in Fig. 4, these differences in prevalence for different patient populations are reflected in the slope of the corresponding equi-PPV (NPV) lines, hence determine a population-specific zone in the ROC space wherein the minimal criteria for PPV (NPV; or PPV and NPV together) are met. This can also have an application in test validation: when the prevalence of disease is known in the validation setting, the zone of successful validation can easily be determined upfront. Upon calculating the risk scores in the validation cohort using the prognostic model under scrutiny, one can then observe whether the ROC curve (or associated 95% confidence interval) is crossing the zone of success. Recently, Willis and Hyde introduced a similar concept, i.e., an “applicable region” in the ROC space. Interestingly, they derived this concept to select studies for meta-analysis as relevant for a certain clinical setting [28, 29]. Evidently, a significant change in application setting, e.g., from secondary care to primary care, will have a more profound impact on case mix, being the distribution of outcomes and predictive factors [30]. Such change of application setting and patient spectrum will be outside the utility scope of equi-PPV (NPV) lines for gauging test performance.

Ideally, the performance of prognostic models should also be assessed in terms of calibration [7, 23], where one will look to compare the observed probabilities with the predicted probabilities [9]. It is interesting to note that by itself, the definition of a cut-off using PPV and NPV does not require prior calibration of the model. This is due to the fact that calibration is done by applying a monotonic transformation to the score. The independence from calibration is illustrated by the fact that calibration does not usually modify ranking and that the ROC curve is based on the ranking of the scores. It is important to mention that calibrated scores and predictive values have different use. The calibration ensures that the test score reflects the likelihood of a test to predict a patient’s chance to develop a condition [31]. The predictive values give the likelihood that a subset of selected patients develops (or not for the NPV) a condition.

In our pre-eclampsia example, we also hypothesised that the explicit consideration of PPV and NPV criteria in test development also allow dissemination of data-rich “omics” experiments in an alternative and possibly more effective way. Rather than searching for a “golden” combination of markers which meets various stakeholder perspectives, often leading to the unattainable requirement to deliver high PPV and high NPV at the same time, the likelihood of finding subsets of markers which answer PPV and NPV criteria independently will increase. In other words, a single “omics” analysis can deliver inputs to two different test paradigms, which can be interpreted independently or conjunctly, depending on the clinical context. As can be seen in Fig. 5, prognostic tests that meet the separate pre-set criteria do not necessarily have very high AUROCs, rather they have skewed risk score distributions, and hence skewed ROC curves.

A limitation of this approach is the possibility that the combined rule-in and rule-out stratification using independent tests can deliver conflicting information: e.g. a patient might be classified to be simultaneous high risk and low risk. One will have to determine what fraction of patients will be in this “conflict” group, and what the appropriate care would be for the patients in this group. Again, this will be depending on the clinical context; for instance, in our case of pre-eclampsia risk stratification in low-risk nulliparous, this group might be considered “unclassified” and stay in the “one-fits-all” care pathway which is the current clinical standard.

Finally, we consider it conceivable that multi-component tests which are developed to comply with either the rule-in or the rule-out test will also be more generalisable. Using the web tool, it was found that models which comply with a (stringent) PPV criterion are characterised by a fraction of cases which are very well discriminated (following a tight risk score distribution in controls). Vice versa, models which comply with a (stringent) NPV criterion are characterised by a fraction of controls which are very well discriminated (following a tight risk score distribution in the cases). Provisional this is not a result of mere overfitting or patient spectrum, it may well be the predictors constituting a good rule-in model are more directly associated with the pathophysiology of the condition (e.g. pre-eclampsia) or its severity. Likewise, a good rule-out model might constitute predictors which are strong determinants of non-disease (or health). If so and arguably, the methods presented here may enhance the transportability of such models across different healthcare and demographic settings. Validation of dedicated rule-in or rule-out models will need to be done to confirm this hypothesis. Of note, we applied a rule-in criterion (PPV≥ 0.20; *S*_*n*_ ≥ 0.50) to develop a biomarker based prognostic model for pre-eclampsia in low-risk nulliparous once before; in that instance, we were able to validate the model as developed in a cohort of New Zealand and Australian women in an European patient population [1].

## Conclusion

The equi-PPV and equi-NPV lines are valuable statistical tools which enrich the well-established ROC analysis to quantify the clinical usefulness of a prognostic test in a simple and meaningful fashion. The enriched ROC plots simultaneously visualise sensitivity, specificity, NPV and/or PPV. They can be used to estimate the clinical relevance of a prognostic test by visualising simultaneously a range of statistics and in particular its rule-in and/or its rule-out performance. It can also be used to compare prognostic tests or to gauge the impact of e.g., prevalence on the predictive performance requirements.

It is of note that equi-PPV and equi-NPV lines are also relevant for the development and evaluation of diagnostic tests.

The reader is invited to explore this feature at the following website: http://d4ta.link/ppvnpv/.

## Additional file

Additional file 1 Expression of predictive values in terms of prevalence and likelihood ratio’s. (DOCX 21 kb)

## Abbreviations

ACOG: American College of Obstetricians and Gynecologists
AUC: Area Under the Curve
AUROC: Area Under Receiver Operating Characteristic
FN: False Negatives
FP: False Positives
FPR: False Positive Rate
NPV: Negative Predictive Value
NPV_c_: Negative Predictive Value cut-off
p: Prevalence
PPV: Positive Predictive Value
PPV_c_: Positive Predictive Value cut-off
ROC: Receiver Operating Characteristic
*S*_*n*_: Sensitivity
*S*_*p*_: Specificity
TN: True Negatives
TP: True Positives
