# Supplementary material for: A novel method for interrogating receiver operating characteristic curves for assessing prognostic tests
Source: Diagn Progn Res. 2017 Nov 15;1:17. doi: 10.1186/s41512-017-0017-y (PMC6460848; doi:10.1186/s41512-017-0017-y)
Supplement: Supplementary file 1 — Expression of predictive values in terms of prevalence and likelihood ratio’s. (DOCX 21 kb) [file 41512_2017_17_MOESM1_ESM.docx]

**EXPRESSION OF PREDICTIVE VALUES IN TERMS OF PREVALENCE AND LIKELIHOOD RATIO’S**

**Expression of PPV in terms of prevalence and positive likelihood ratio**

With positive likelihood ratio LR_(+)_, being expressed in function of sensitivity S_n_ and specificity S_p_ as follows:

$${LR}_{(+)}=\frac{S_{n}}{\left( 1-S_{p} \right)}$$

Equation 3:

$$PPV=\frac{S_{n} p}{S_{n} p+\left( 1-S_{p} \right) \left( 1-p \right)} \left( 3 \right)$$

can be rearranged as such that a linear relationship between the multiplicative inverse of PPV, prevalence p and positive likelihood ratio LR_(+)_ becomes apparent:

$${PPV}^{-1}={{LR}_{(+)}}^{-1}\frac{1-p}{p}+1 (3^{'})$$

Or

$${PPV}^{-1}=p^{-1}\frac{1}{{LR}_{(+)}}+\frac{{LR}_{(+)}-1}{{LR}_{(+)}} (3'')$$

From equation 3’’ it is clear that for a given LR_(+)_, the PPV will increase with increasing prevalence.

**Expression of NPV in terms of prevalence and negative likelihood ratio**

With Negative likelihood ratio LR_(-)_, being expressed in function of sensitivity S_n_ and specificity S_p_ as follows:

$${LR}_{(-)}=\frac{{1-S}_{p}}{\left( S_{n} \right)}$$

equation 4:

$$NPV=\frac{S_{p} (1-p)}{\left( 1-S_{n} \right) p+S_{p} (1-p)} \left( 4 \right)$$

can be rearranged as such that a linear relationship between the multiplicative inverse of NPV, prevalence p and negative likelihood ratio LR_(-)_ becomes apparent:

$${NPV}^{-1}={LR}_{(-)}\frac{p}{1-p}+1 (4^{'})$$

or

$${NPV}^{-1}={(1-p)}^{-1}{LR}_{(-)}+1-{LR}_{\left( - \right)} (4")$$

From equation 4’’ it is clear that for a given LR_(-)_, the NPV will decrease with increasing prevalence.
